# Quantifying myelin in crossing fibers using diffusion‐prepared phase imaging: Theory and simulations

**DOI:** 10.1002/mrm.28907

**Published:** 2021-07-13

**Authors:** Michiel Cottaar, Wenchuan Wu, Benjamin C. Tendler, Zoltan Nagy, Karla Miller, Saad Jbabdi

**Affiliations:** ^1^ Wellcome Centre for Integrative Neuroimaging—Centre for Functional Magnetic Resonance Imaging of the Brain John Radcliffe Hospital University of Oxford Oxford United Kingdom; ^2^ Laboratory for Social and Neural Systems Research University of Zurich Zurich Switzerland

**Keywords:** diffusion MRI, magnetic susceptibility, myelin, phase imaging, white matter

## Abstract

**Purpose:**

Myelin has long been the target of neuroimaging research. However, most available techniques can only provide a voxel‐averaged estimate of myelin content. In the human brain, white matter fiber pathways connecting different brain areas and carrying different functions often cross each other in the same voxel. A measure that can differentiate the degree of myelination of crossing fibers would provide a more specific marker of myelination.

**Theory and Methods:**

One MRI signal property that is sensitive to myelin is the phase accumulation. This sensitivity is used by measuring the phase accumulation of the signal remaining after diffusion‐weighting, which is called diffusion‐prepared phase imaging (DIPPI). Including diffusion‐weighting before estimating the phase accumulation has two distinct advantages for estimating the degree of myelination: (1) It increases the relative contribution of intra‐axonal water, whose phase is related linearly to the thickness of the surrounding myelin (in particular the log *g*‐ratio); and (2) it gives directional information, which can be used to distinguish between crossing fibers. Here the DIPPI sequence is described, an approach is proposed to estimate the log *g*‐ratio, and simulations are used and DIPPI data acquired in an isotropic phantom to quantify other sources of phase accumulation.

**Results:**

The expected bias is estimated in the log *g*‐ratio for reasonable in vivo acquisition parameters caused by eddy currents (~4%‐10%), remaining extra‐axonal signal (~15%), and gradients in the bulk off‐resonance field (<10% for most of the brain).

**Conclusion:**

This new sequence may provide a *g*‐ratio estimate per fiber population crossing within a voxel.

## 1 INTRODUCTION

Myelin is one of the main constituents of the brain’s white matter^1^ and plays a key role in modulating the speed of action potentials in axons.^2^, ^3^ The degree of myelination has been shown to change over the lifespan^4^ with different white matter tracts myelinating at different stages during childhood.^5^, ^6^ Activity‐dependent changes in myelination have also been found in adults.^7^ The amount of myelin typically decreases during aging and has been found to be altered in a variety of pathologies,^4^ such as leukodystrophies, multiple sclerosis,^8^ and schizophrenia.^9^ Accordingly, producing accurate in vivo maps of myelin content has been a longstanding goal in brain imaging.

A common metric to quantify the degree of myelination is the *g*‐ratio, which is defined as the inner over the outer radii of the myelin sheath.^2^ Using multiple MRI modalities one can obtain an estimate of the average voxel‐wise *g*‐ratio in a voxel in vivo by combining measurements of myelin and axonal volume fractions.^10^, ^11^, ^12^, ^13^ The axonal volume fraction can be estimated from diffusion MRI, using a multicompartment fit to the diffusion‐weighted signal.^14^, ^15^, ^16^, ^17^, ^18^ A wide variety of different MRI modalities have been proposed to estimate the myelin volume fraction.^19^, ^20^ Most of these rely on directly imaging the myelin water, which can be distinguished from the rest of the water based on its short T_2_ using multiecho spin‐echo sequences,^21^, ^22^, ^23^ its short $T_{2}^{*}$ using multiecho gradient‐echo sequences,^24^, ^25^ its short T_1_ using an inversion‐recovery sequence,^26^ or based on magnetization transfer between the myelin macromolecules and water.^27^

The interpretability of estimating the *g*‐ratio from volume fractions is limited, as it only gives an average *g*‐ratio per voxel. It is an average across both myelinated and unmyelinated axons^28^ because the method assumes that all axons have the same *g*‐ratio.^11^ It is also an average across fiber populations in voxels where multiple fibers cross each other, which is a common configuration in the human brain.^29^, ^30^ Furthermore, this approach relies on the accuracy of the volume fraction estimates,^31^ which has been questioned for both the axonal volume fractions^32^ and the myelin volume fractions.^13^, ^19^, ^20^ Here we aim to overcome these limitations by proposing a novel sequence, which is directly sensitive to the *g*‐ratio (rather than the volume fractions) and allows to distinguish between crossing fibers.

Diffusion‐weighting gradients can be used to distinguish between crossing fibers. Diffusion‐weighting has previously been combined with all of the myelin‐sensitive metrics listed above to obtain tract‐specific metrics, namely T_2_,^33^, ^34^ $T_{2}^{*}$,^35^, ^36^ T_1_,^35^, ^37^ and magnetization transfer.^38^ Unfortunately, diffusion‐weighted gradients take such a long time to build up this sensitivity to fiber orientation that there will be very little signal left associated with the myelin water because of its short T_2_.^39^ Rather, after diffusion‐weighting, the signal mainly comes from water relatively distant from the myelin, which reduces the sensitivity of the relaxation and magnetization transfer properties to myelin.

On the other hand, the off‐resonance magnetic field generated by the myelin magnetic susceptibility not only affects the local myelin water, but also has an effect throughout the intra‐ and extra‐axonal spaces in nearby tissue.^40^, ^41^, ^42^, ^43^, ^44^ This provides a means to detect the properties of myelin from more long‐lived T_2_ species still visible after diffusion‐weighting. Hence, we propose a sequence called diffusion‐prepared phase imaging (DIPPI), where we estimate the myelin‐induced phase accumulation in the MR signal still visible after diffusion‐weighting.

In this work, we first derive how the phase accumulation measured by DIPPI is related to the *g*‐ratio in crossing fiber bundles. We then use simulations and data in an isotropic phantom to show under which conditions we can reliably estimate the myelin‐induced phase accumulation and hence the *g*‐ratio from DIPPI, despite many potential confounds, namely eddy currents, nonmyelin sources of susceptibility, and remaining signal from extra‐axonal water after diffusion‐weighting.

## 2 THEORY

### 2.1 Overview

**FIGURE 1:**
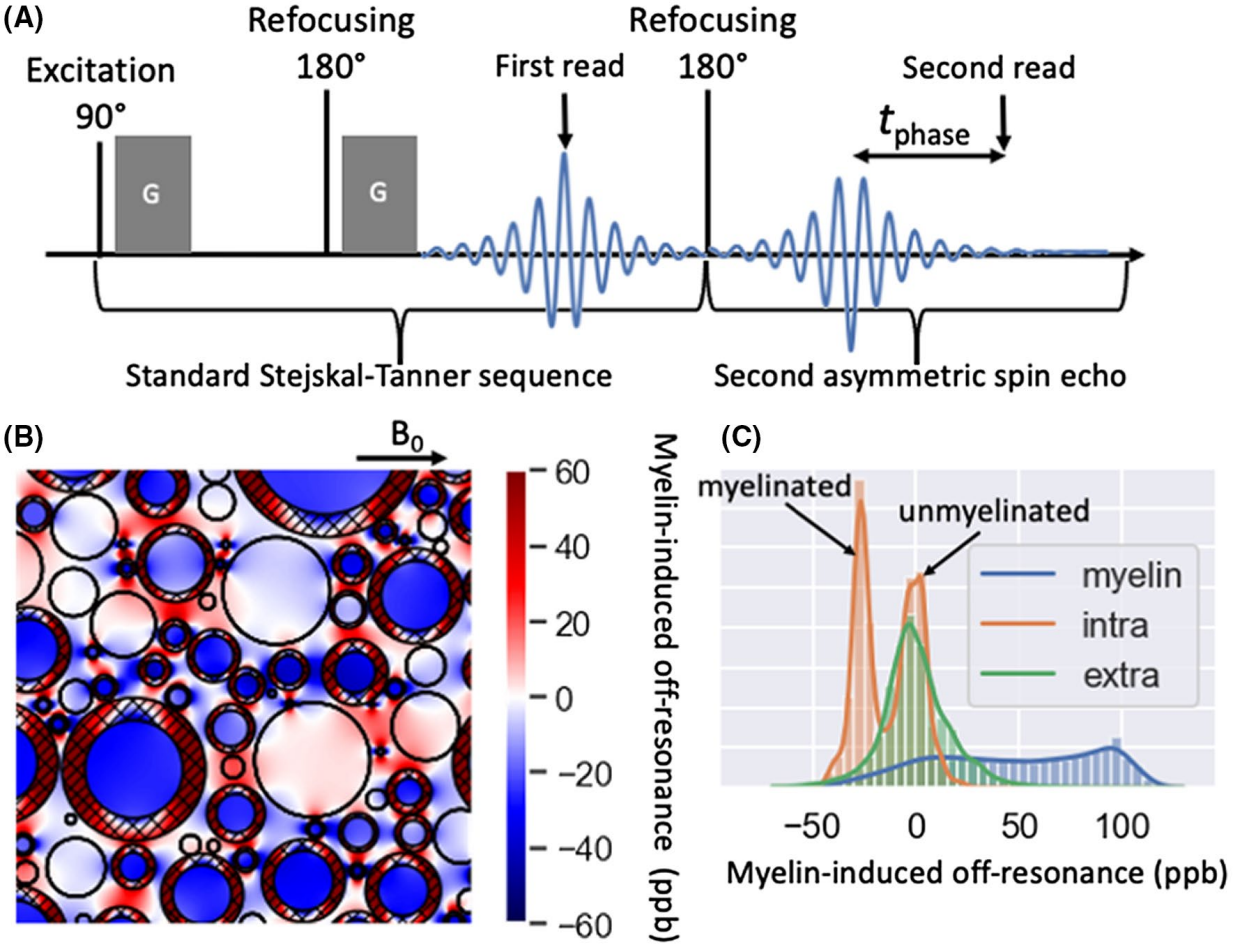
A, Proposed diffusion‐prepared phase imaging (DIPPI) sequence to measure the off‐resonance frequency of diffusion‐weighted water. The sequence consists of a standard Stejskal‐Tanner sequence followed by a second echo‐planar imaging readout in an asymmetric spin echo. B, Illustration of white matter with axons as parallel cylinders, some of which are myelinated (myelin sheaths are hashed). Overlaid is the off‐resonance field induced by the myelin according to the hollow‐cylinder model.^40^ C, The distribution of the field is shown in B in the intra‐axonal (orange), extra‐axonal (green), and myelin (blue) compartments. After diffusion‐weighting the signal will be dominated by the intra‐axonal water in axons perpendicular to the diffusion‐weighting gradient. For this intra‐axonal water, the off‐resonance frequency has a bimodal distribution corresponding to the unmyelinated and myelinated axons with the latter having an off‐resonance frequency proportional to the log *g*‐ratio

The DIPPI sequence consists of a standard diffusion‐weighted spin‐echo sequence to which we have added an additional refocusing pulse and readout. The acquisition window of the second readout is offset from the second spin echo by a tuneable delay, which we refer to as the phase accumulation time $t_{\text{phase}}$ (Figure 1A). The phase difference between these two readouts allows us to estimate the off‐resonance frequency of the water still visible after diffusion‐weighting without being confounded by any phase accumulation during the diffusion‐weighting.

Combining diffusion‐weighting with phase imaging provides two advantages for measuring the degree of myelination of individual tracts. First, it increases the relative contribution of the intra‐axonal water to the final signal, particularly at high *b* values.^45^ This has the advantage that while the myelin‐induced magnetic field offset has a complicated spatial profile in the extra‐axonal and myelin space (Figure 1B,C), it is uniform within the intra‐axonal space. For a simplified model of myelinated axons as infinite cylinders, this myelin‐induced off‐resonance frequency in the intra‐axonal space ($\omega_{\text{myelin}}$) is given by:^40^

(1) $$\omega_{\text{myelin}}=‐\frac{3}{4}\omega_{0}\chi_{A}\log g\;\sin^{2}\theta ,$$

where $\omega_{0}$ and $\chi_{A}$ are constants (respectively, the Larmor frequency and the anisotropic component of the myelin susceptibility) and θ is the angle between the fibers and the main magnetic field, which we estimate using the magnitude data from DIPPI. We will assume here that the signal is dominated purely by the intra‐axonal water, although we will investigate the bias that this assumption might induce because of the remaining extra‐axonal signal. In the absence of diffusion‐weighting, contributions from all compartments would need to be considered when fitting the signal.^46^ The second advantage of using diffusion‐weighting is that it adds directional information, which allows us to measure the relative degree of myelination (ie, log *g*‐ratio) between crossing fibers rather than a voxel‐wide average.

An additional feature of DIPPI is that we can also exploit the bimodal distribution of the intra‐axonal off‐resonance frequency (Figure 1C) to fit a two‐population model to data acquired with multiple phase‐accumulation times ($t_{\text{phase}}$). Though for a single $t_{\text{phase}}$, we can obtain the average log *g*‐ratio across both the myelinated and unmyelinated axons, the two‐population model allows us to estimate their relative signal fractions, as well as the average log *g*‐ratio of the myelinated axons.

To explain the analysis, we split it into three parts. First, we estimate the susceptibility‐induced off‐resonance frequency of diffusion‐weighted water taking into account other sources of phase accumulation (ie, movement during the diffusion encoding and eddy currents). Then we discuss how to subtract out the off‐resonance frequency caused by susceptibility sources other than myelin. Finally, we relate the myelin‐induced off‐resonance frequency to the average log *g*‐ratio of crossing fibers.

### 2.2 Estimating the off‐resonance frequency

The DIPPI signal is modulated by both the diffusion‐weighting gradients (ie, the b value and orientation $\hat{g}$) and the phase accumulation time $t_{\text{phase}}$. For each set of *b* values, gradient orientations, and $t_{\text{phase}}$, we acquire two images, one during the initial spin‐echo readout ($S_{\text{SE}}$) and one during the second asymmetric spin‐echo readout ($S_{\text{ASE}}$). In this work, we assume that all data have been acquired with a single *b* value (in addition to b=0 scans), although the model can be extended to multiple *b* values by fitting all parameters independently at each *b* value, except for the fiber orientations and degree of myelination.

For a single *t*_phase_, the expected signal across multiple gradient orientations is given by:

(2) $$S_{\text{SE}}\left(b,\hat{g}\right)=\sum_{k}A_{\text{SE},k}e^{‐b\Delta D_{k}{\left(\hat{g}\cdot {\hat{n}}_{k}\right)}^{2}}e^{i\phi_{\text{SE}}},$$

(3) $$S_{\text{ASE}}\left(t_{\text{phase}},b,\hat{g}\right)=\sum_{k}A_{\text{ASE},k}e^{‐b\Delta D_{k}{\left(\hat{g}\cdot {\hat{n}}_{k}\right)}^{2}}e^{i\left(\phi_{\text{SE}}+\Delta \phi_{\text{eddy}}+\phi_{\text{susc;k}}\right)},$$

where we sum the signal contributions from multiple crossing fiber populations k in an effort to estimate the phase caused by the off‐resonance frequency associated with each fiber population $\phi_{\text{susc;k}}$. The other terms are explained below.

**FIGURE 2:**
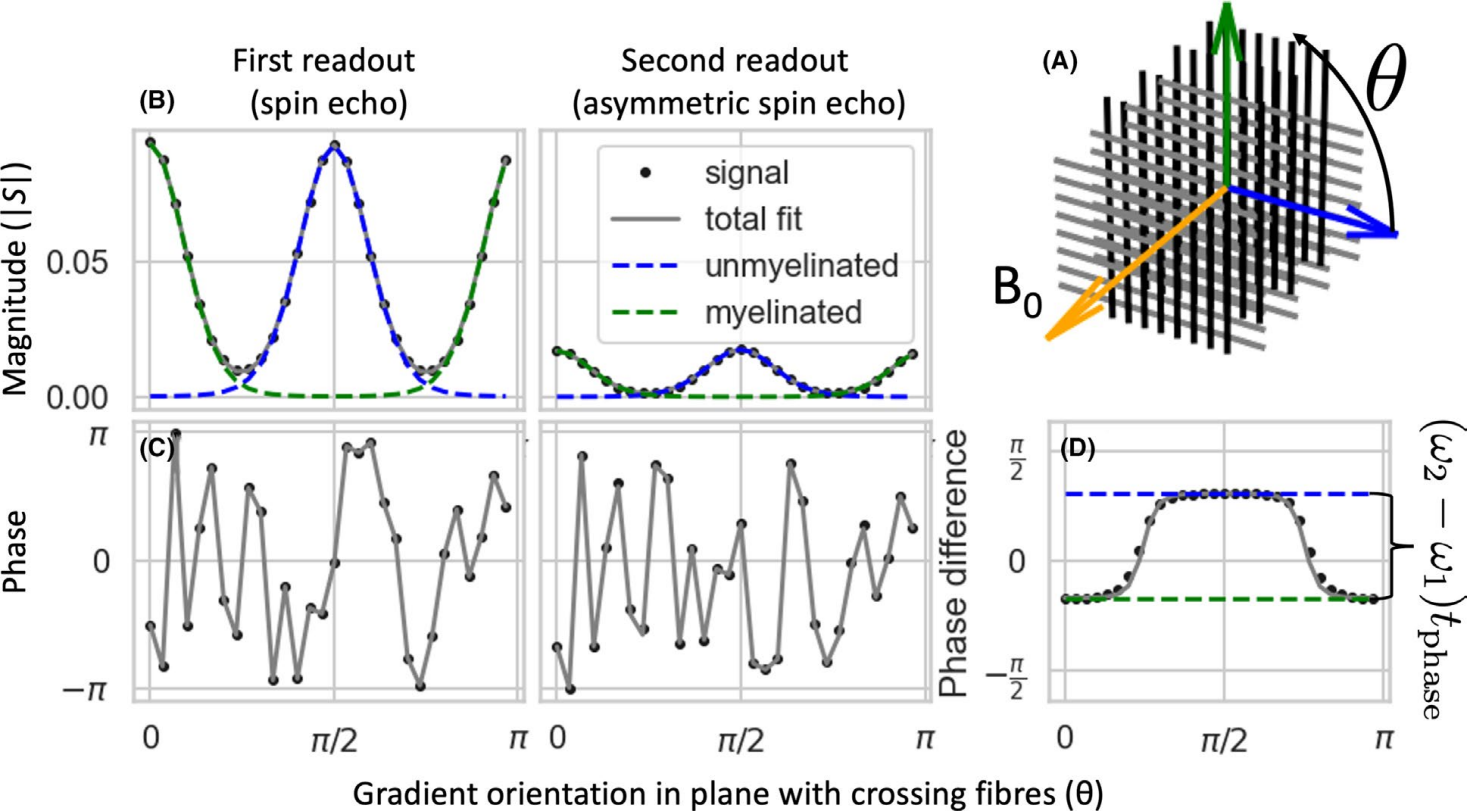
Illustration of the signal estimated from Monte Carlo simulations of two fiber populations (one fully myelinated with a *g*‐ratio of 0.7 and one fully unmyelinated) crossing at right angles and perpendicular to the main magnetic field (A). For ease of illustration, we only consider gradient orientations in the plane of the crossing fibers, but the same principle holds for a three‐dimensional (3D) acquisition. The magnitude is fitted as a sum of 2 Gaussians (Watson distributions in 3D), which have maxima perpendicular to the fiber orientation (B). These Gaussians will have a much lower amplitude in the second readout, but are assumed to have the same width between the readouts. Although the phase will be different for each gradient orientation because of movement during the diffusion‐weighting (C), the phase difference between the two readouts still provides an estimate of the difference in susceptibility‐induced off‐resonance frequency of the two fiber populations (D)

The first part of these equations (ie, $A_{\text{ASE/SE},k}e^{‐b\Delta D_{k}{\left(\hat{g}\cdot {\hat{n}}_{k}\right)}^{2}}$) is concerned with the magnitude of the image (Figure 2B). As we are mainly interested in the phase, we fit to the magnitude the simplest model that can distinguish between crossing fibers, namely one where the signal profile for each crossing fiber is given by a Watson distribution with an amplitude $A_{k}$ and width $\Delta D_{k}$. This is the signal profile expected if the signal for each fiber population can be modeled by an axisymmetric diffusion tensor with eigenvalues $\lambda_{\|,k}$ and $\lambda_{⊥,k}$ and volume fraction $f_{k}$. In that case, the amplitude corresponds to $A_{k}=S_{0}f_{k}e^{‐b\lambda_{⊥,k}}$ and the width to $\Delta D_{k}=\lambda_{\|,k}‐\lambda_{⊥,k}$.

The width of these Watson distributions ($\Delta D_{k}$) only depends on the diffusion‐weighting; hence, it should be the same for both the symmetric and asymmetric spin echoes. The signal amplitudes ($A_{k}$), on the other hand, will decrease over time based on $T_{2}$ and $T_{2}^{\prime }$ dephasing, which means that we will have a different amplitude for each readout: $A_{\text{SE},k}$ and $A_{\text{ASE},k}$. Using multiple phase‐accumulation times, it is possible to use the dependence of $A_{\text{ASE},k}$ on $t_{\text{phase}}$ to estimate both the $T_{2,k}$ and $T_{2,k}^{\prime }$ of the diffusion‐weighted signal for each fiber population.

The phase accumulation before the first readout will be affected by many factors, such as eddy currents or movement during the diffusion encoding larger than a few tens of micrometers. As such movements are unavoidable in in vivo MRI, we simply consider the phase at the first readout to be a random number that has to be estimated independently for each volume ($\phi_{\text{SE}}$). Our interest here is in the phase accumulation between the two readouts, which is induced by the off‐resonance frequency of any eddy currents ($\Delta \phi_{\text{eddy}}$) and the brain’s susceptibility ($\phi_{\text{susc}}$; Figure 2C,D).

### 2.3 Eddy‐current–induced off‐resonance frequency

Eddy currents caused by the strong diffusion gradients introduce a phase offset that is dependent on the gradient amplitude and orientation. Here, we are interested in the contribution of eddy currents to the phase accumulated between the two readouts ($\Delta \phi_{\text{eddy}}$). We model this phase offset using spherical harmonics:

(4) $$\Delta \phi_{\text{eddy}}\left(\hat{g},t_{\text{phase}}\right)=\sum_{l=0}^{l_{\text{max}}}\sum_{m=‐l}^{l}c_{\mathrm{lm}}\left(t_{\text{phase}}\right)Y_{l}^{m}\left(\hat{g}\right),$$

where $Y_{l}^{m}$ are the spherical harmonic functions mapping the parameters $c_{\mathrm{lm}}$ onto the sphere. Because the eddy currents decay over time following the diffusion‐weighting gradients, we cannot simply model these parameters using a linear equation as we will for the susceptibility below.

We can only estimate the odd‐order spherical harmonics (which are asymmetric), but not the even‐order spherical harmonics (which are symmetric and hence degenerate with the susceptibility‐induced phase offsets). Fortunately, the dominant component of the eddy current‐induced phase offset is asymmetric as we will confirm in the Results section.

One exception, where we can estimate part of the even‐order components of the eddy current‐induced phase offset, is if we acquire a shell with $t_{\text{phase}}=0$ (ie, both readouts are at their respective spin echoes). For this shell, the susceptibility‐induced phase offset is zero, so we can attribute any phase accumulated between the two readouts to the eddy currents and hence estimate the even components of $c_{\mathrm{lm}}\left(t_{\text{phase}}=0\right)$. Then, rather than assuming that the even‐order components of $c_{\mathrm{lm}}\left(t_{\text{phase}}\right)$ are zero, we can instead model them by assuming they match $c_{\mathrm{lm}}\left(t_{\text{phase}}=0\right)$. This corrects for any eddy current‐induced phase accumulation between the spin echoes, although it still cannot correct for the evolution of the even components of the spherical harmonics during the phase accumulation time.

### 2.4 Correcting for the nonmyelin susceptibility

The susceptibility‐induced off‐resonance frequency is not only influenced by the local myelin ($\omega_{\text{myelin}}$), but also by many other sources of susceptibility ($\omega_{\text{bulk}}$):

(5) $$\phi_{\text{susc;k}}=\left(\omega_{\text{myelin,k}}+\omega_{\text{bulk}}\right)t_{\text{phase}}$$

These other sources of susceptibility include both distant sources (eg, the air–tissue interface) and other local sources of susceptibility (eg, blood vessels). To resolve between these myelin and nonmyelin susceptibilities, we make the assumption that any nonmyelin source of susceptibility (ie, $\omega_{\text{bulk}}$) is equal for all crossing fibers. This allows us to estimate the myelin‐induced frequency offset difference between crossing fibers (with indices *k* and *k*′) as:

(6) $$\omega_{\text{myelin;k}}‐\omega_{\text{myelin,}k^{\prime }}=\frac{\phi_{\text{susc,k}}‐\phi_{\text{susc,}k^{\prime }}}{t_{\text{phase}}}$$

This assumption is most accurate if the crossing fibers overlap spatially (ie, they interdigitate). On the other hand, if the crossing fibers are on opposite sides of a voxel, their off‐resonance frequency may differ based on any large‐scale magnetic field gradients or differences in local susceptibility field (eg, one fiber population being closer to blood vessels).

Equation 6 only gives the difference in the myelin‐induced frequency offset between crossing fibers, which would only allow one to estimate the difference in myelination between crossing fibers. To obtain an absolute estimate of the *g*‐ratio for each individual fiber, we need additional information. This can be obtained by changing the head orientation, which modulates the relation between the off‐resonance frequency $\omega_{\text{myelin}}$ and the *g*‐ratio (Equation 1). Once the frequency offset (Equation 1) has been estimated for multiple head orientations, the individual *g*‐ratios can be obtained through linear regression.

### 2.5 Estimating the *g*‐ratio

One additional obstacle to estimating the *g*‐ratio is that although there is a simple linear relationship between the myelin‐induced off‐resonance frequency and the *g*‐ratio within each axon (Equation 1), each fiber population consists of many axons with potentially varying *g*‐ratios. We propose two methods to still obtain a meaningful estimate of the *g*‐ratio. Both methods assume that the signal after diffusion‐weighting is dominated by the intra‐axonal water, for which the myelin‐induced phase evolution is given by the hollow fiber model (Equation 1).^40^

The first method is only valid for $t_{\text{phase}}$ short enough that the signal phase from the most myelinated axons is still in rough alignment with the signal phase from the least myelinated axons (ie, the unmyelinated axons with a *g*‐ratio of 1 and hence $\omega_{\text{myelin}}=0$). In this limit, the myelin‐induced phase accumulation is determined by the average of the off‐resonance frequency in each axon (weighted by its signal contribution). Hence we have:

(7) $$\omega_{\text{myelin,k}}=‐\frac{3}{4}\omega_{0}\chi_{A}{\langle \log g\rangle }_{k}\sin^{2}\theta_{k},$$

where ${\langle \log g\rangle }_{k}$ is the signal‐weighted average log *g*‐ratio of the fiber population *k* across both myelinated and unmyelinated axons.

For longer $t_{\text{phase}}$, this simple relation above no longer holds and we need to adopt a two‐compartment model: the myelinated and unmyelinated fibers (Figure 1C). For the myelinated fibers we assume that the *g*‐ratios are sufficiently similar that we can characterize this population based solely on their average log *g‐*ratio. Hildebrand and Hahn^47^ found a range of *g*‐ratios from 0.6 up to 0.75 in the spinal cord of various mammals. Because this is quite a narrow range compared with the *g*‐ratio of 1 for unmyelinated fibers, we expect the two‐compartment model to be adequate for any reasonable $t_{\text{phase}}$ (at least in healthy tissue). For this two‐compartment model, we expect a phase evolution of:

(8) $$\omega_{\text{myelin},k}t_{\text{phase}}=\text{angle}\left(\left(1‐f_{\text{myelin};k}\right)+f_{\text{myelin};k}e^{‐i\frac{3}{4}\omega_{0}\chi_{A}{\langle \log g\rangle }_{\text{myelin},k}\sin^{2}\theta_{k}t_{\text{phase}}}\right),$$

where $f_{\text{myelin;k}}$ is the relative signal fraction of myelinated versus unmyelinated axons, ${\langle \log g\rangle }_{\text{myelin};k}$ is the average log *g*‐ratio of just the myelinated axons, and “angle” is a function that returns the angle of a complex number. Equation 7 is the first‐order Taylor expansion of Equation 8 with the average log *g*‐ratio across all axons defined as ${\langle \log g\rangle }_{k}=f_{\text{myelin;k}}{\langle \log g\rangle }_{\text{myelin};k}$.

**FIGURE 3:**
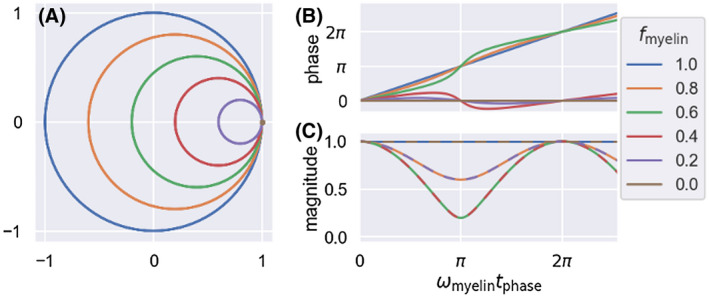
Signal evolution over time for the sum of unmyelinated axonal water (ω=0) and myelinated axonal water ($\omega =\omega_{\text{myelin}}$). Each line shows the evolution for a different signal fraction of myelinated axons ($f_{\text{myelin}}$; color coded according to legend on the right). A, Shows the signal evolution through complex signal space with B and C showing just the phase or magnitude evolution. For only myelinated axons ($f_{\text{myelin}}=1$ in blue) the signal traces a circle in complex space with constant magnitude and linearly increasing phase. As the fraction of unmyelinated axons increases, the size of this circle shrinks and importantly it no longer centers on the origin, which leads to a nonlinear phase and magnitude evolution

The phase evolution of the signal phase according to Equation 8 is shown in Figure 3. At small $t_{\text{phase}}$, the phase evolution is approximately linear with a slope of $f_{\text{myelin}}\omega_{\text{myelin}}$. However, as $\omega_{\text{myelin}}t_{\text{phase}}$ approaches π the phase starts to approach the phase within just the dominant population (ie, unmyelinated axons for $0<f_{\text{myelin}}<0.5$ or myelinated axons for $0.5<f_{\text{myelin}}<1$; Figure 3B). By combining data across multiple $t_{\text{phase}}$, we can capture this time‐dependent nonlinear phase evolution to characterize both the fraction of myelinated axons ($f_{\text{myelin;k}}$) and their average log *g*‐ratio (${\langle \log g\rangle }_{\text{myelin;k}}$) for each crossing fiber population. The evolution of the magnitude also contains information on the myelination (Figure 3C), but in practice, this will be very hard to disentangle from other sources of $T_{2}^{\prime }$ dephasing, which we do not consider here. For this reason, we will constrain the myelination purely on the phase and not the magnitude information.

We directly fit all parameters describing the magnitude and phase (Equations 2 and 3) to each voxel of complex DIPPI data. An overview of the full model with a description of all model parameters is given in the Supporting Information S1 with a summary of which parameters can be estimated for different acquisition schemes in Supporting Information Table S1. Parameters are optimized using local optimization after a multistep initialization process to prevent the parameter optimization from getting stuck in local minima Supporting Information S2.

## 3 METHODS

### 3.1 Sensitivity to bias in off‐resonance frequency

Any difference in the off‐resonance frequency between the crossing fibers not attributable to the myelin (δω) will lead to a bias in the log *g*‐ratio (δlogg). We can quantify this bias using Equation 1 (for $\chi_{A}=‐100\;\text{ppb}$)^40^:

(9) $$\delta \log g=\frac{4\delta \omega }{3\omega_{0}\chi_{A}\sin^{2}\theta }\approx 0.045\frac{1}{\sin^{2}\theta }\frac{\delta \omega }{\text{Hz}}\frac{7T}{B_{0}}$$

The sensitivity of logg on any error in the off‐resonance frequency depends on the angle with the main magnetic field, with the sensitivity going to infinity for fibers parallel to the main magnetic field ($\sin^{2}\theta =0$).

To quantify the frequency of such fiber orientations, we compute the $\sin^{2}\theta$ for two randomly oriented fiber populations and investigate the distribution of the $\sin^{2}\theta$ for the fiber population that happens to be more perpendicular or that more parallel to the main magnetic field. These simulations were run using either any two random fiber orientations or excluding any fiber orientations with a crossing angle lower than 45°.

### 3.2 Phantom scan

The DIPPI sequence was implemented on a 7T Siemens scanner. To validate the sequence and to characterize the influence of eddy currents, we scanned an isotropic oil phantom. Because the phantom is isotropic, we attribute any variation in the signal phase between different gradient orientations to eddy currents, which allows direct estimation of their contribution. Three axial slices were acquired using the sequence shown in Figure 1A with the following scan parameters: image resolution 2 mm × 2 mm, slice thickness 2 mm, field of view 192 mm × 192 mm, 6/8 partial Fourier, 10‐mm slice gap, echo spacing 0.81 ms, *b* value 2 ms/µm^2^. Sixty diffusion directions and their reverse were acquired (ie, 120 diffusion‐weighted images in total) as well as 8 b=0 volumes. The effective echo times for the two readouts were 81 and 165 ms, respectively ($t_{\text{phase}}=30$ ms). After phase unwrapping across gradient orientations (described in the Supporting Information S3), the phase offset observed in the b=0 images was subtracted. Spherical harmonics were then fitted to the phase to estimate the $c_{\mathrm{lm}}$ in our eddy current model (Equation 4). We also directly compare the phase offset for two near‐orthogonal gradient orientations either without any corrections, after subtracting out all odd‐order spherical harmonics, or after subtracting out both the odd‐order spherical harmonics and the even‐order spherical harmonics estimated for $t_{\text{phase}}=0$.

### 3.3 Reference susceptibility‐weighted imaging

To quantify the magnitude of the off‐resonance field including all sources of susceptibility, we used publicly available phase imaging data from the QSM reconstruction challenge in Graz.^48^ This data set was acquired from a healthy volunteer using a wave‐CAIPI sequence^49^ with an isotropic resolution of 1.05 mm and echo time of 25 ms on a 3T MRI scanner. The provided data have already been phase unwrapped. We convert the phase image to frequency by dividing it by the echo time. Then we compute the magnitude of the local frequency gradient. This gradient gives a rough idea of how different the off‐resonance field might be for fiber populations on opposite sides of a voxel.

### 3.4 Simulations to test extra‐axonal contribution

The proposed model assumes that any remaining signal after diffusion‐weighting is intra‐axonal. To investigate potential biases caused by any extra‐axonal signal remaining, we ran Monte Carlo simulations using Camino’s datasynth^50^ of crossing fibers using the default diffusivity of 2 µm^2^/ms. Fibers were crossing at 90° (in the x‐ and y‐directions) with both being perpendicular to the main magnetic field (in the z‐direction). All axons were modeled as perfect cylinders in the x‐direction or y‐direction organized in interleaving single‐axon–thick planes (Figure 2A). The distance between planes was fixed to 1 µm. By varying the outer axonal diameter between 0.5 µm and 0.98 µm, we vary the extra‐axonal volume fraction from 0.25 to 0.8. Within each plane, half of the axons were myelinated (g=0.7), with the other half being unmyelinated. The trajectory of 100,000 simulated spins was output.

The spin evolution over the sequence including the effect of the myelin susceptibility was modeled for a 7T scanner at multiple different *b* values. The myelin‐induced off‐resonance frequency was modeled according to the hollow‐fiber model^40^ with myelin susceptibility of $\chi_{I}=‐100\text{ppb}$ (isotropic component) and $\chi_{A}=‐100\text{ppb}$ (anisotropic component). In this model, the off‐resonance field at every point is evaluated as the contribution of the surrounding axon’s myelin (if any) given by Equation 1 and the sum of the dipole‐like extra‐axonal field of all other axons. The simulated data were fit using the procedure described in Supporting Information S2 to estimate the bias due to the signal contribution from extra‐axonal water. The confounds of eddy currents, non‐myelin contributions to the susceptibility, and measurement noise were not included in these simulations. In addition, the myelin water itself was not explicitly modeled as its contribution is expected to be very small due to its short T_2_ (in fact the border between the intra‐ and extra‐axonal water was infinitely thin and non‐permeable in the simulations).

### 3.5 Simulations to test degeneracy between parameters

Finally, we model and then fit DIPPI data using the model described in the Theory section to investigate any degeneracies between parameter estimates. In these simulations, the initial amplitudes and signal widths are set assuming a stick‐like diffusion model ($d_{\|}=1.7\frac{\text{ms}}{{\mu m}^{2}}$), the phase at the first readout ($\phi_{\text{SE}}$) is set to a random value between 0 and 2π for each scan and the off‐resonance frequency caused by nonmyelin susceptibility ($\omega_{\text{other}}$) is set to a random large value (so that the phase wraps many times between each $t_{\text{phase}}$). The l=1 components of the eddy currents are computed from $a+bt_{\text{phase}}$, where *a* and *b* are random numbers drawn from Gaussian distributions $N\left(0,\sigma =1.4\text{rad}\right)$ and $N\left(0,\sigma =18\text{Hz}\right)$, respectively. We set $T_{2}=60$ ms^51^ and $T_{2}^{*}=35$ ms^25^ as appropriate for 7T. We consider two crossing fibers at 90°, both of which have 50% of the axons being myelinated with a *g*‐ratio of 0.7. Either both fiber populations are perpendicular to the main magnetic field or one is parallel and the other is perpendicular to the main magnetic field. We also consider the case where we have data for both of these fiber configurations with respect to the main magnetic field, which is an extreme case of what can be achieved by scanning with multiple head orientations.

To quantify how large the head rotations need to be to resolve the degeneracy between the *g*‐ratio of the crossing fibers, we treat it as a linear regression problem. In the case of two crossing fibers (labeled k and k’), DIPPI provides for each head orientation an estimate of the g‐ratio difference weighted by $\sin^{2}\theta$: ${\langle \log g\rangle }_{k}\sin^{2}\theta_{k}‐{\langle \log g\rangle }_{k^{\prime }}\sin^{2}\theta_{k}$ with some uncertainty σ. We simulate four different head orientations, namely rest [${\hat{B}}_{0}=\left(0,0,1\right)$], forward [${\hat{B}}_{0}=\left(0,‐s,c\right)$], right [${\hat{B}}_{0}=\left(‐s,0,c\right)$], and left [${\hat{B}}_{0}=\left(s,0,c\right)$], where s=sinϕ and c=cosϕ for a head rotation of ϕ, which for simplicity we assume is the same in each direction. We also assume a uniform prior in the log *g* between log 0.6 and log 1, which has a standard deviation of ($\frac{‐\log0.6}{\sqrt{12}}$). We compute the uncertainty on the best‐fit log *g* from these linear equations as a function of the head rotation ϕ, the uncertainty per head orientation σ, and the two fiber orientations.

## 4 RESULTS

### 4.1 Sensitivity to bias in off‐resonance frequency

**FIGURE 4:**
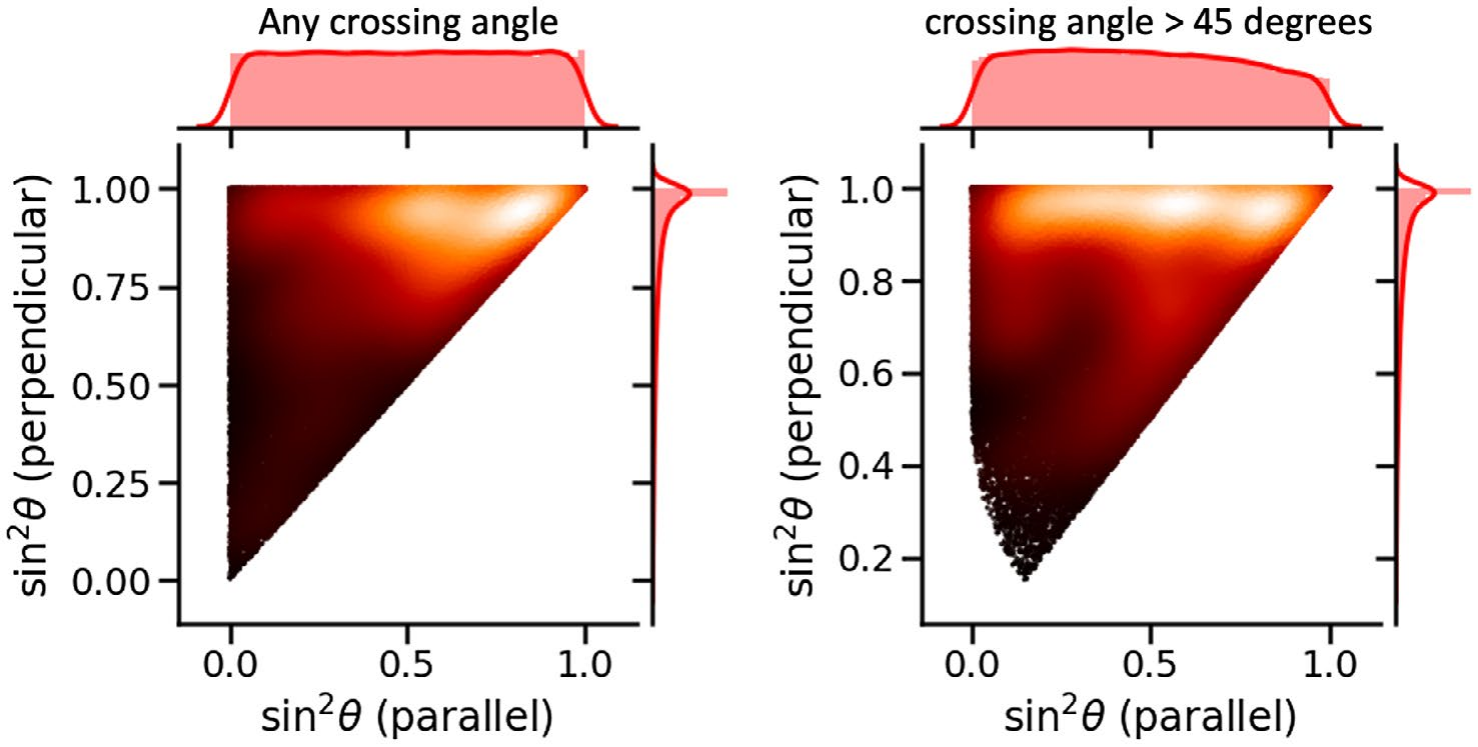
Distribution of the $\sin^{2}\theta$ for two randomly oriented crossing fibers, where θ is the angle with the main magnetic field. The y‐axis shows the distribution for whichever of the two fibers happens to be more perpendicular to the main magnetic field and the x‐axis the distribution for the more parallel fiber. The left panel shows the distribution for any two random fibers, whereas on the right we exclude any configurations with an angle between the crossing fibers lower than 45°. Lighter colors indicate higher density

The effect of errors in the off‐resonance frequency on the estimated log *g*‐ratios is modulated by $\sin^{‐2}\theta$ (Equation 9). If we consider the fiber orientations in the brain random with respect to the main magnetic field, we find that in regions of two crossing fibers typically one of them is close to being perpendicular to the main magnetic field with $\sin^{2}\theta \approx 1$ (y‐axis in Figure 4). The log *g* estimate for this fiber orientation is relatively insensitive to errors in the off‐resonance frequency. The other fiber population has a roughly flat distribution in $\sin^{2}\theta$ (x‐axis in Figure 4), which means that although in some voxels it too might be relatively insensitive to noise, in other voxels this fiber orientation may be close to parallel to the main magnetic field ($\sin^{2}\theta \approx 0$), which means that even a small deviation in the off‐resonance frequency will cause a large error in the estimated *g*‐ratio. To convert biases in the off‐resonance frequency to errors in the estimated *g*‐ratios, we will assume perpendicular fibers ($\sin^{2}\theta =1$) in the data in the section below; however, it should be noted that at least for some fiber populations the induced error in the *g*‐ratio will be a lot higher.

### 4.2 Bias caused by eddy currents

**FIGURE 5:**
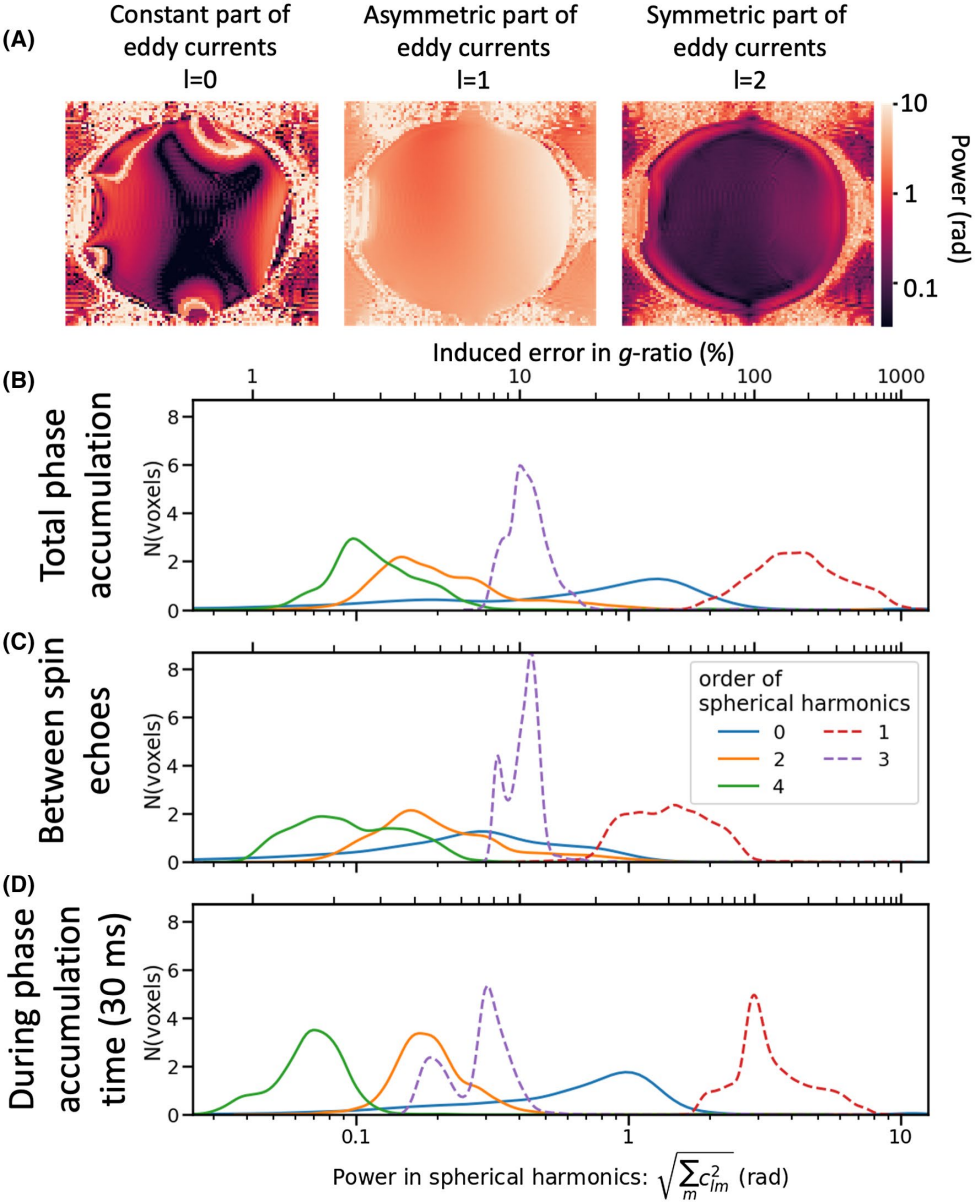
Phase accumulation between the two readouts caused by nonzero eddy currents measured in an isotropic oil phantom at b=2 ms/µm^2^ for a phase accumulation time *t_phase_* of 30 ms on a 7T Siemens scanner. The phase accumulation measured at *b* = 0 is subtracted out (to subtract out the susceptibility field); then spherical harmonics are fitted to the eddy current‐induced field offset. A, Map of the power in these spherical harmonics. B, Histogram of the maps shown in A. Dashed lines show the odd components (that can be corrected for); solid lines show the even components (that cannot be corrected for). C, Phase accumulation between the first and second spin echoes (measured using *t_phase_* of 0 ms). D, Phase accumulation in the 30 ms between the second spin echo and the readout. The x‐axis in B–D is given in both the power in the spherical harmonics in radians (bottom) and the relative error that this angular offset will induce in the estimated *g*‐ratio for fibers perpendicular to the main magnetic field from Equation 9 as a percentile (top)

To investigate the potential bias because of eddy current‐induced phase accumulation, we compare the expected myelin‐induced phase offsets with the phase offsets induced by eddy currents found in an isotropic phantom on a 7T scanner. The eddy current‐induced phase offset is dominated by the l=1 components of the spherical harmonics (Figure 5A). Fortunately, these and the other odd‐order components can be estimated even when scanning an anisotropic medium like the brain’s white matter. The even components of the spherical harmonics (for l≠0) are more problematic as they are degenerate with the myelin‐induced frequency offset between crossing fibers. Fortunately, the power in the even components is typically much smaller than in the odd components (Figure 5B). Still the estimated l=2 component could cause a bias in the *g*‐ratio in fibers perpendicular to the main magnetic field of a few percentile. At the edge of the phantom, this induced bias exceeds 10%. This large l=2 component at the edge of the phantom accumulates mostly between the two spin echoes (Figure 5C), rather than between the second spin echo and the readout (Figure 5D). This suggests that it might be better to approximate the l=2 component in a separate scan with $t_{\text{phase}}=0$ rather than ignoring it altogether.

Unexpectedly, despite subtracting out the phase offset caused by the B0 field (estimated using the b=0 scans) the l=0 component is still substantial (Figure 5B). This means that after averaging out all gradient orientations there is a net phase offset on the order of 0.5 rad to 1 rad between the b=0 and b=2 ms/µm^2^ scans. The origin of this component is unclear; however, we note that it is swamped by the size of the B0 field (discussed below) and will not cause a bias in the frequency offset measured between different fiber orientations.

**FIGURE 6:**
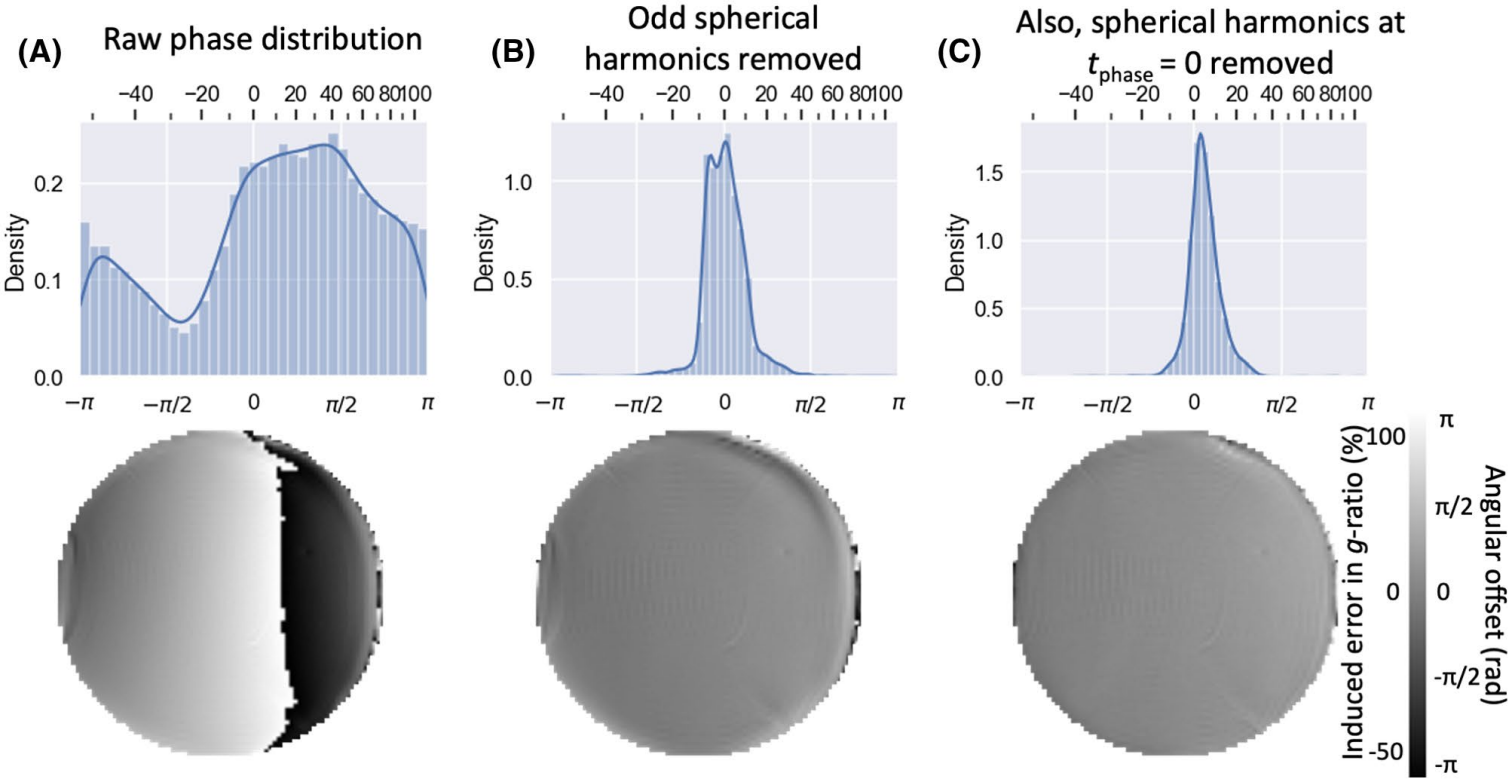
Distribution of difference in the eddy current‐induced phase offset between two roughly orthogonal gradient orientations without correction (A), after correcting only the odd‐order spherical harmonics (B), or after also subtracting the even‐order spherical harmonics estimated at $t_{\text{phase}}=0$ (C). Top panels show the histogram across all three slices. Bottom panels show the phase map for the center slice. The x‐axis in the top panel and color bar in the bottom panels show both the angular offset and the relative error this would induce in the *g*‐ratio (in percentile) for fibers perpendicular to the main magnetic field. In all panels, this figure shows the phase difference at $t_{\text{phase}}=30$ ms for an isotropic phantom in a 7T scanner

Figure 6 illustrates the result of the correction of the eddy‐induced phase offset between two nearly orthogonal gradient orientations. Without correcting for eddy currents, there is a substantial phase offset, which would hide any myelin‐induced phase offsets (Figure 6A). Subtracting out the odd‐order spherical harmonics gets rid of most of the eddy current‐induced phase (Figure 6B). The remaining phase deviations, particularly at the edge of the phantom, are caused by the large size of the l=2 spherical harmonic components (Figure 5A) and can be further reduced by subtracting out the phase difference accumulated at $t_{\text{phase}}=0$ (Figure 6C). The histogram of the resulting phase offset does not nicely center at zero, which would in this case correspond to a bias in the *g*‐ratio estimated for fibers perpendicular to the main magnetic field of approximately 4%.

### 4.3 Bias caused by bulk susceptibility

**FIGURE 7:**
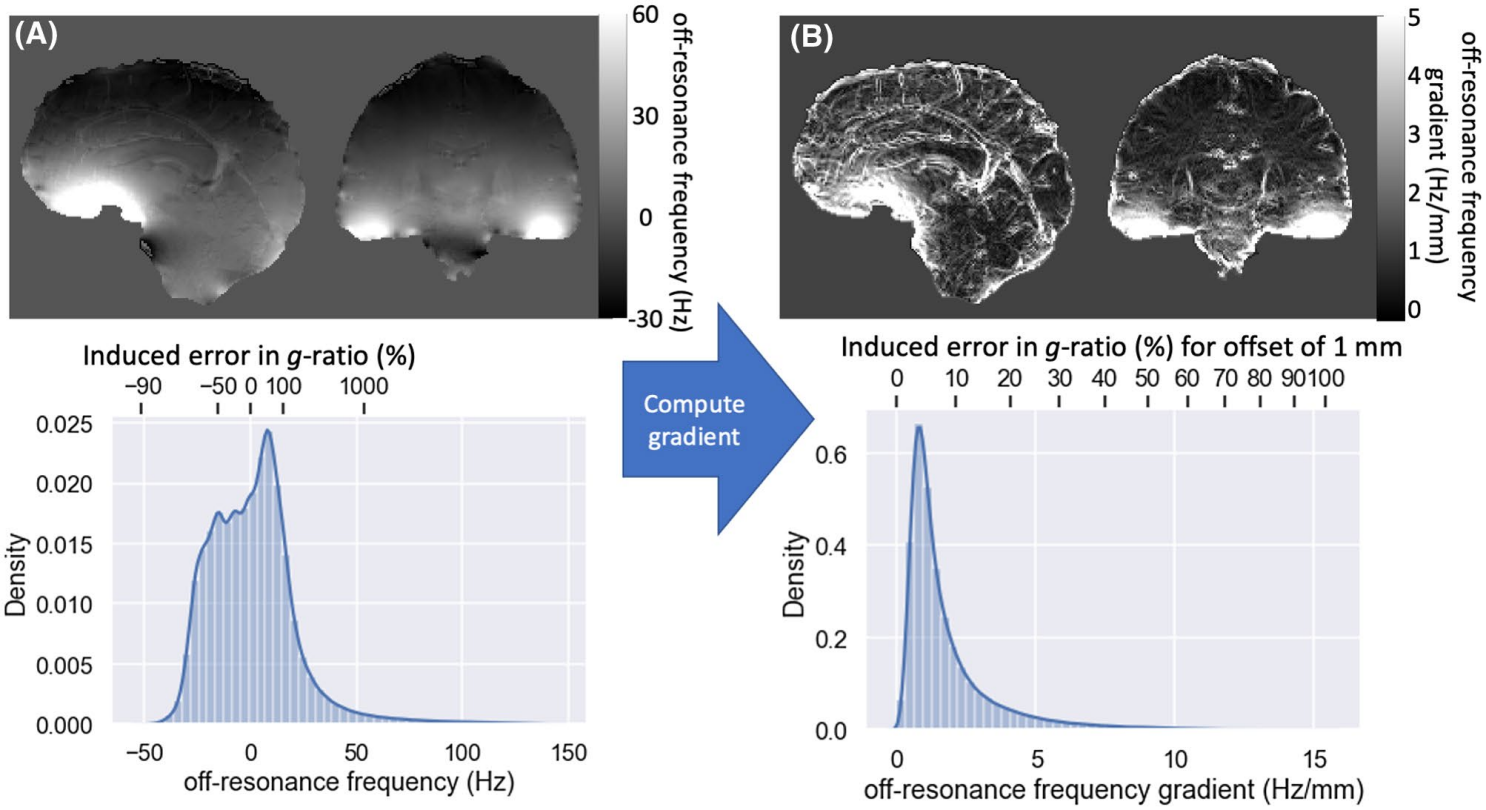
A, Off‐resonance frequency distribution estimated for a healthy volunteer at echo time = 25 ms on a 3T scanner (after phase unwrapping). B, Distribution of the gradients in the off‐resonance field. The x‐axis in the histogram shows both the off‐resonance frequency (gradient) at the bottom and the error this would induce in the *g*‐ratio in percentile for perpendicular gradients. On the right, this error would be incurred for crossing‐fiber population 1‐mm apart in the direction of the maximum gradient

The large‐scale background off‐resonance frequency field is much larger than the expected myelin‐induced frequency offset (Figure 7A). This field can bias the *g*‐ratio estimate if crossing fibers do not interdigitate, but are actually on opposite sides of the same voxel. In such a case, they may have different contributions from the large‐scale off‐resonance field. To estimate the size of this effect, we computed the spatial gradient of the off‐resonance frequency field (Figure 7B). For most of the brain, the gradient of this field is so small that even if the crossing fibers were on the opposite side of a voxel (ie, ~1‐mm apart), the resulting frequency offset would bias the *g*‐ratio by approximately 5% for fibers perpendicular to the main magnetic field. However, close to the major arteries or the air–brain interface (eg, the orbitofrontal regions), the gradient of the off‐resonance frequency becomes large enough to bias the estimated *g*‐ratios by over 10% to 20% in the case that the crossing fibers are not interdigitated.

### 4.4 Bias caused by extra‐axonal water signal

**FIGURE 8:**
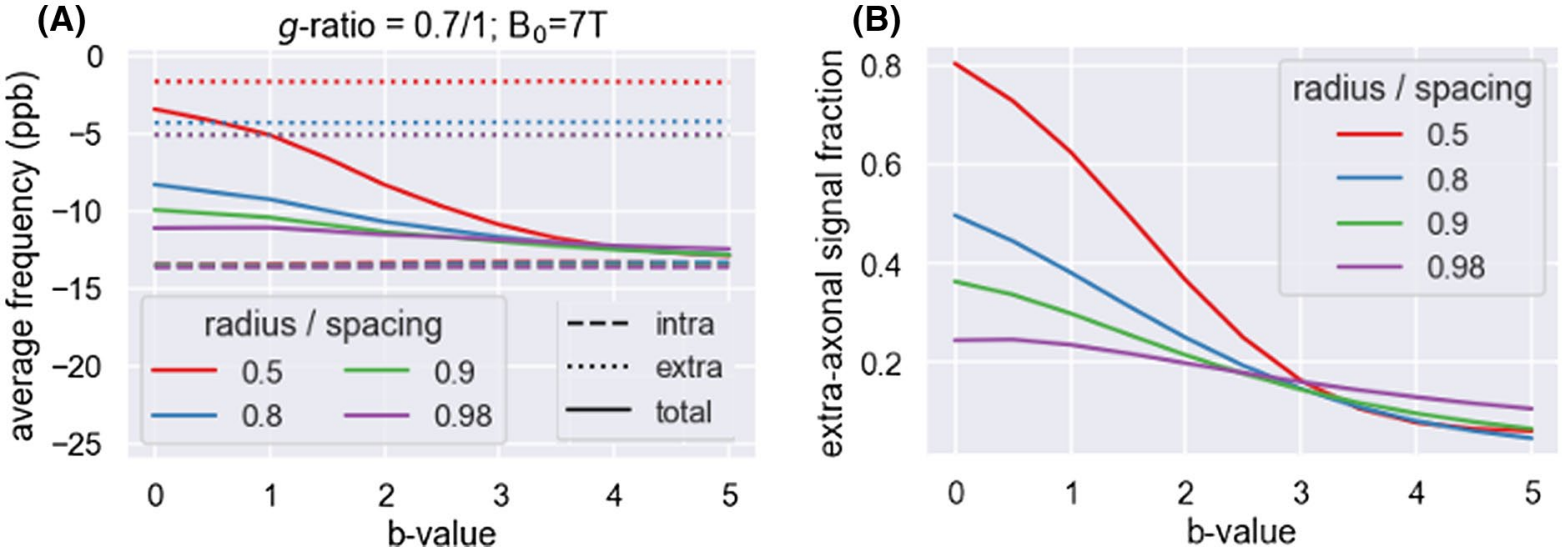
Bias on the off‐resonance frequency caused by extra‐axonal water. A, Average myelin‐induced off‐resonance frequency in Camino Monte Carlo simulations of infinitely long crossing cylinders (half are unmyelinated and half have *g* = 0.7) with different spacings (see color legend). As the *b* value increases, the off‐resonance frequency of the total signal (solid line) approaches that of the intra‐axonal water (dashed), although some bias to the extra‐axonal frequency (dotted) remains. B, This approach is caused by the decrease in the extra‐axonal signal fraction with *b* value in these Monte Carlo simulations

Finally, bias in the estimated parameters can also come from the remaining contribution of the extra‐axonal water even after diffusion‐weighting. For reasonable *b* values (~3 ms/µm^2^), we find that the actual off‐resonance frequency is approximately 10% to 20% smaller than expected for pure intra‐axonal water (Figure 8A), which would lead to a similar underestimation in the logg. This underestimation is caused by the approximately 15% extra‐axonal signal contribution remaining at *b* = 3 ms/µm^2^ (Figure 8B), modulated by the average off‐resonance frequency of the extra‐axonal water (Figure 8A). Interestingly, in the simulations the 15% extra‐axonal signal contribution was consistent across a wide variety of different axonal densities (color scales). The fiber packing configuration will affect both the average extra‐axonal off‐resonance frequency^41^, ^52^ and how fast the extra‐axonal signal decays with *b* value. The simulations here use an unrealistic fiber configuration of perfectly straight cylinders crossing each other at right angles in a perfect grid, which means that the bias found here is only a rough estimate of the bias size expected in real tissue.

### 4.5 Degeneracies between fitted parameters

**FIGURE 9:**
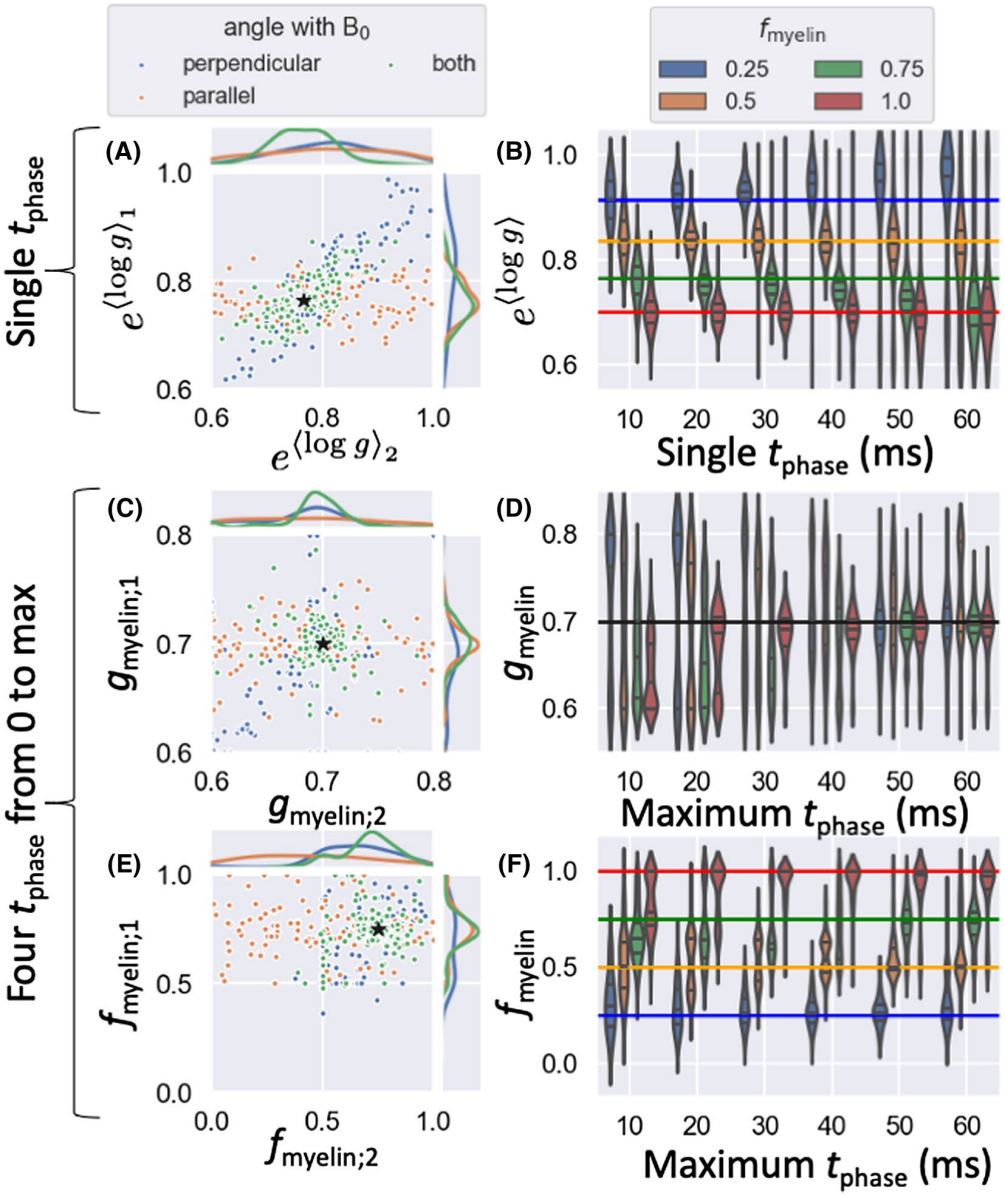
Results for fitting the model with various head orientations (A,C,E) or different phase‐accumulation times *t*_phase_ (B,D,F). We either consider data with a single *t*_phase_ (20 ms in A), where we estimate the average log *g*‐ratio across both myelinated and unmyelinated fibers (A,B), or data with four different *t*_phase_ uniformly distributed from 0 to the maximum *t*_phase_ (60 ms in C and E) for which we estimate the average log *g*‐ratio of the myelinated axons ($g_{\text{myelin}}$) and the fraction of myelinated axons ($f_{\text{myelin}}$). In the left column (A,C,E), each dot represents the estimated value for one of 100 different noise iterations for the case where both fibers are perpendicular to the main magnetic field (blue), one of the fibers is parallel, and the other is perpendicular to the main magnetic field (orange); or we have multiple head orientations combining the information from the first two (green). The ground truth value is given by the black star. The right column (B,D,F) shows the distribution of these individual estimates as a function of the maximum *t*_phase_ for various values of the fraction of myelinated axons (*f*_myelin_) for the case of multiple head orientations (green in the left column)

While Although the eddy currents, gradients in the nonmyelin susceptibility, and extra‐axonal water all might bias the estimated *g*‐ratios as discussed above, a more fundamental limitation arises because we can only estimate the difference in the myelin‐induced frequency between crossing fibers. In case of data only acquired with a single head orientation and single $t_{\text{phase}}$, we can only estimate a weighted difference in log *g* between two crossing fibers [$\langle \log g_{1}\rangle \sin^{2}\theta_{1}‐\langle \log g_{2}\rangle \sin^{2}\theta_{2}$]. If both fibers have the same angle with the main magnetic field (ie, $\theta_{1}=\theta_{2}$), this implies we can estimate the difference in logg between the crossing fibers, not what the $\langle \log g_{1}\rangle$ and $\langle \log g_{2}\rangle$ actually are. This case is illustrated in Figure 9A by the distributions of blue dots, which all have a very similar $\langle \log g_{1}\rangle ‐\langle \log g_{2}\rangle$, even though the individual estimates of logg are unconstrained. On the other hand, if fibers have different angles with the main magnetic field, we are less sensitive to the logg that is more parallel to the main magnetic field, which changes the slope of the degeneracy (ie, the line along which the points lie in Figure 9A). The most extreme case of this is when one fiber population is parallel to the main magnetic field (eg,$\sin\theta_{2}=0$), in which case we are completely insensitive to the myelination of that population [ie, $\langle \log g_{2}\rangle$], but can estimate the $\langle \log g\rangle$ of the other population (orange in Figure 9A). By combining information across multiple head orientations, we can constrain the *g*‐ratios of the crossing fibers as the intersection between the different degenerate solutions (green in Figure 9A).

Figure 9A considers the case where we aim to estimate the average log *g* across both myelinated and unmyelinated axons (Equation 7) using a single *t*_phase_. However, as this *t*_phase_ becomes too long, the signal from the unmyelinated and myelinated axons become out of phase with each other, and the resulting phase approaches that of the dominant component (Figure 3B). As a result, the estimated average log *g* will then match that of the dominant component (ie, the myelinated axons if $f_{\text{myelin}}>0.5$ or the unmyelinated axons if $f_{\text{myelin}}<0.5$; Figure 9B). When we have data across multiple *t*_phase_, we can exploit this behavior to estimate both the average log *g* across both components and the log *g* of the dominant component, which allows the estimation of both the fraction and *g*‐ratio of myelinated axons (Figure 9D,F). In Figure 9D,F, we see that this technique works best if the signal is dominated by myelinated axons (ie, large $f_{\text{myelin}}$), although as long as the $t_{\text{phase}}$ is long enough the $f_{\text{myelin}}$, and to a lesser extent the $g_{\text{myelin}}$, can be estimated even when the unmyelinated compartment dominates ($f_{\text{myelin}}=0.25$, blue‐violin plots).

The nonlinear time evolution of the myelin‐induced phase offset can also be exploited to distinguish it from the nonmyelin susceptibility. This leads to reasonable fits to the fraction and *g*‐ratio of myelinated axons, even if data were only acquired with a single head orientation (blue in Figure 9C,E). With multiple head orientations, these estimates are still substantially improved (green in Figure 9C,E).

**FIGURE 10:**
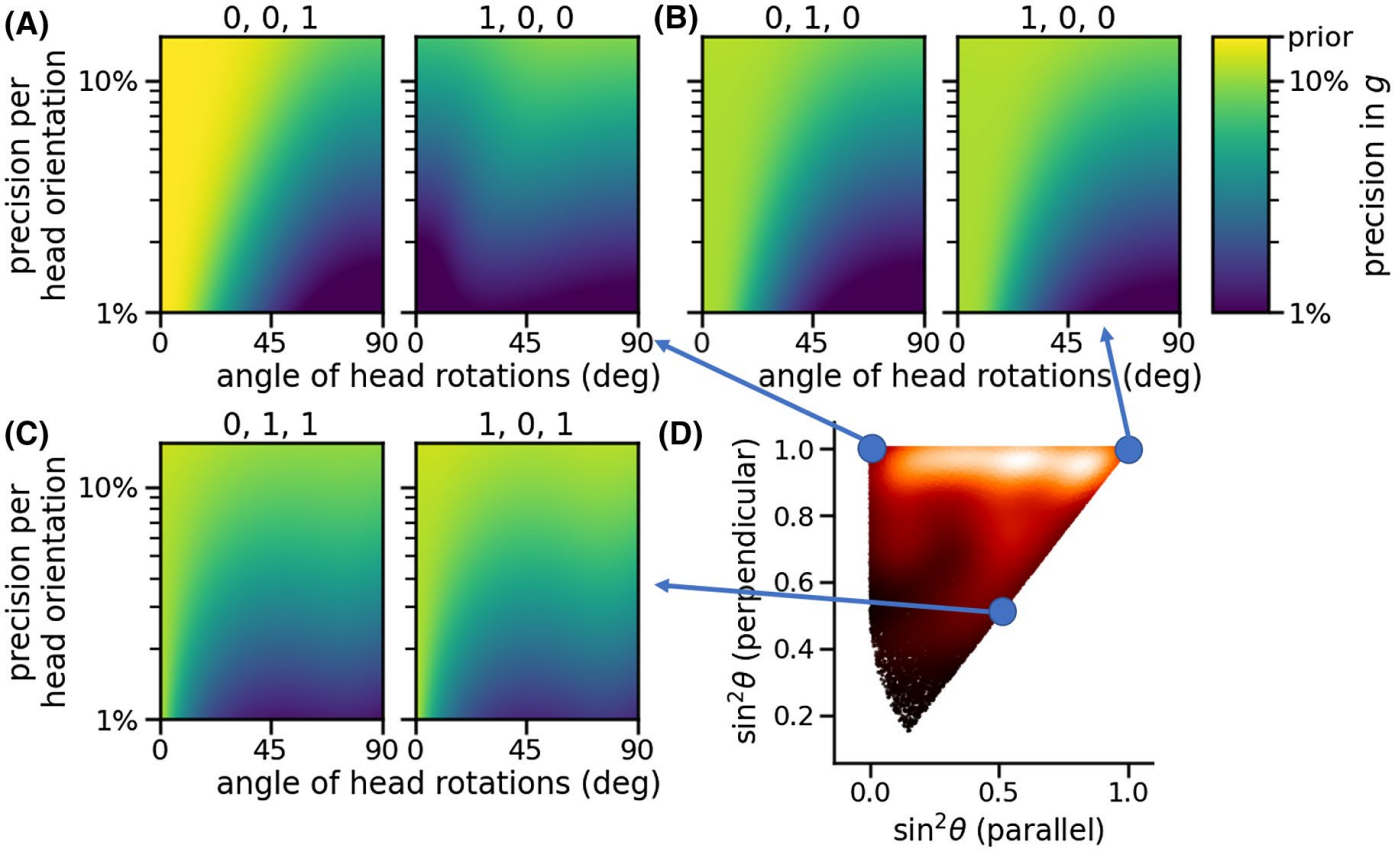
A‐C, Precision with which the log *g*‐ratio can be estimated across multiple head orientations for three sets of crossing fibers. The color coding in each panel shows the precision (ie, standard deviation) of the estimated log *g*‐ratio when data are combined across head orientations. This final precision is a function of the precision with which the difference in $\sin^{2}\theta$‐weighted log *g* can be estimated for every single head orientation on the y‐axis and the angle between the different head orientations on the x‐axis. Four head positions were considered (rest, forward, left, right) as described in the Methods section. Fiber orientation is given above each panel in (x, y, z) with the main magnetic field pointing in the z‐direction in the rest head position. D, Shows where these crossing fibers fit in the distribution of crossing fiber orientations with the main magnetic field from Figure 4. A yellow color indicates that the precision in the estimated log *g* is similar to the prior of 15.9% (ie, the data did not constrain the log *g* in a meaningful way)

Figure 10 illustrates the precision with which the log *g*‐ratio can be estimated as a function of the crossing fiber orientations, angle of the head orientations, and precision with which $\langle \log g_{1}\rangle \sin^{2}\theta_{1}‐\langle \log g_{2}\rangle \sin^{2}\theta_{2}$ can be estimated for each head orientation. Without substantial head rotations the *g‐*ratio of fibers parallel to the main magnetic field is very poorly constrained by the data (left in Figure 10A); however, we are able to estimate the *g*‐ratio for any crossing fibers (right in Figure 10A). This is the equivalent to the orange dots in Figure 9A. For two fibers crossing perpendicular to the main magnetic field, we can estimate only the difference in the log *g*‐ratio, which does little to actually constrain the individual *g*‐ratios even if measured with a very high precision (Figure 10B or blue dots in Figure 9A). Substantial head rotations on the order of 20° are required to break this degeneracy in the individual log‐*g* estimates. As the fibers are not perfectly perpendicular to the main magnetic field, the head rotation requirement to break this degeneracy decreases with head rotations on the order of 10° being sufficient for fibers with a 45° angle to the main magnetic field (Figure 10C).

## 5 DISCUSSION

We propose a sequence, DIPPI, to estimate the *g*‐ratio of axons within the white matter by measuring the off‐resonance frequency of the water remaining visible after diffusion‐weighting. After diffusion‐weighting, the signal is dominated by intra‐axonal water in axons that run perpendicular to the diffusion gradient orientation. We exploit the linear relationship between the log *g*‐ratio and the myelin‐induced frequency offset in this intra‐axonal water (Equation 1) to estimate the *g*‐ratio after correcting for several other sources of off‐resonance frequency. DIPPI allows one to go beyond the voxel‐wise average estimates of *g*‐ratio to get an estimate of the average log *g*‐ratio in every fiber population. For a single, short $t_{\text{phase}}$, this log *g*‐ratio is averaged across both myelinated and unmyelinated axons. Simulations suggest that at 7T a $t_{\text{phase}}$of approximately 20 to 30 ms should allow for sufficient phase accumulation to be robustly observed (Figure 9B) without significant biases caused by nonlinearities expected as the phase diverges between the myelinated and unmyelinated axons (Figure 3). By varying $t_{\text{phase}}$, we can separate the myelinated and unmyelinated axons to estimate the volume fraction and *g*‐ratio of the myelinated axons in each crossing‐fiber population. Robust estimation of the *g*‐ratio of myelinated axons may require a maximum $t_{\text{phase}}$ above approximately 50 ms at 7T (Figure 9D,F). Similar phase accumulations can be achieved on a 3T scanner by multiplying the phase‐accumulation times by $\frac{7}{3}$.

Many other sources can affect the amount of phase accumulated in the diffusion‐weighted signal besides the myelin susceptibility. In DIPPI, we measure the phase accumulation between the two readouts after diffusion‐weighting, which is unaffected by any phase accumulated during the diffusion‐weighting (Figure 2). However, phase accumulation between these readouts is still affected by remaining extra‐axonal signal, eddy currents, and nonmyelin sources of susceptibility.

In Monte Carlo simulations with greatly simplified geometries, we found that the remaining extra‐axonal water signal at *b* = 3 ms/µm^2^ is approximately 15% (Figure 8B). To first order, this extra‐axonal water has a similar myelin‐induced frequency as the water within unmyelinated axons, which suggests that this would lead to an overestimation of the fraction of unmyelinated axons (and a corresponding bias in the average log *g*‐ratio; Figure 8A).

The phase accumulated because of eddy currents can be mostly corrected for by modeling them as depending linearly on the gradient orientations. However, not all higher‐order terms can be so easily corrected. DIPPI data in an isotropic phantom (7T scanner with *b* = 2 ms/µm^2^) suggest these higher‐order terms might bias our *g*‐ratio estimates up to approximately 4% to10% (Figure 6).

Given sufficient scanning time, these simulations suggest that acquiring a *b* value of 3 ms/µm^2^ or higher might be worthwhile to minimize the bias incurred from the extra‐axonal water signal. The robustness of the results at such a high *b* value might depend on adopting eddy‐nulled diffusion‐weighting sequences to minimize the bias induced by the eddy currents. For more limited scan times, a lower *b* value can be used to get more robust, albeit biased, *g*‐ratio estimates.

The off‐resonance field generated by any nonmyelin sources of susceptibility is generally much larger than that generated by myelin (Figure 7A). As long as the crossing fibers interdigitate, we can assume that the nonmyelin susceptibility contributes equally to their nonresonance fields, which allows us to estimate the difference in myelin‐induced susceptibility between the crossing fibers. If the fibers do not interdigitate, but are instead 1‐mm apart, this assumption could lead to a substantial bias, especially close to the air–brain tissue boundary and major arteries (Figure 7B). Crucially, the fiber tracts do not have to interdigitate at the level of individual axons as long as the size of the individual fascicles is small compared with the voxel size. This interdigitation assumption is likely to hold in the center of white matter tracts, where the axonal density can be reasonably assumed to not be strongly biased to one side of the voxel. Hence, when analyzing crossing fibers in regions with strong gradients in the off‐resonance frequency (eg, the orbitofrontal cortex), it would be prudent to limit oneself to the center of the white matter tracts.

Even if fibers interdigitate, the large size of the nonmyelin‐induced field still means that we can only estimate the difference in myelination between crossing fibers. For data with multiple‐head orientations, we can get around this limitation to get an estimate of the myelination for each crossing‐fiber population (Figure 10). However, this technique will not work in single‐fiber regions. There are alternative approaches that do not require multiple‐head orientations. Background field removal might be sufficient to remove $\omega_{\text{bulk}}$ under the assumption that the local susceptibility is dominated by myelin,^53^, ^54^, ^55^ or the bulk off‐resonance frequency might be estimated from the off‐resonance in the extra‐axonal water by combining data across multiple *b* values with different extra‐axonal contributions. Alternatively, the curvature of white matter tracts naturally varies the angle between the fiber orientation and the main magnetic field, which we can exploit under the assumption that the fiber myelination is constant along the tract. The reliability of these various approaches will be investigated in future work.

When fitting the two‐pool model to estimate both the fraction and *g*‐ratio of the myelinated axons, additional sources of bias might occur. These estimates rely on the time dependence of the off‐resonance frequency caused by the difference in off‐resonance frequency between myelinated and unmyelinated axons (Figure 3). Hence, the estimates will be biased by any other sources of time dependence in the off‐resonance frequency, which could arise by having multiple compartments with different $T_{2}$ and off‐resonance frequency. However, we are unaware of any evidence for such time dependence in the off‐resonance frequency at these long echo times.

Finally, we note that there are substantial uncertainties in our estimates of the anisotropic component of the myelin susceptibility ($\chi_{A}$)^43^ limiting the accuracy of the resulting *g*‐ratio. A reliable estimate of this constant is crucial to accurately map the *g*‐ratio to the intra‐axonal myelin‐induced frequency offset (Equation 1).

The combination of theory, simulations, and phantom data presented here suggests that DIPPI may be able to obtain a reliable measure of the *g*‐ratio in crossing fibers. We plan to further explore this using both in vivo and ex vivo data in future work.

## Supporting information


**TEXT S1** Model summary
**TEXT S2** Parameter fitting
**TEXT S3** Unwrapping the phase on a sphere
**TABLE S1** Acquisition requirements for the parameters of interest
