# Supplementary material for: Quantifying myelin in crossing fibers using diffusion‐prepared phase imaging: Theory and simulations
Source: Magn Reson Med. 2021 Jul 13;86(5):2618–34. doi: 10.1002/mrm.28907 (PMC8581995; doi:10.1002/mrm.28907)
Supplement: Supplementary file 1 — TEXT S1 Model summary TEXT S2 Parameter fitting TEXT S3 Unwrapping the phase on a sphere TABLE S1 Acquisition requirements for the parameters of interest [file MRM-86-2618-s001.docx]

# Supplementary Materials

## S1: Model summary

For clarity we provide here a summary of the full model fitted to the complex signal of the first spin echo (SE) readout and the second asymmetric spin echo (ASE) readout:

$S_{\text{SE}}\left( b, \hat{g} \right)= e^{i\phi_{\text{SE}}}\sum_{k} A_{\text{SE},k}e^{-b{\Delta D}_{k}\left( \hat{g}\cdot\hat{n}_{k} \right)^{2}},$ (10)

$S_{\text{ASE}}\left( t_{\text{phase}},b,\hat{g} \right)=e^{i\left( \phi_{\text{SE}}+\Delta\phi_{\text{eddy}}+\omega_{\text{bulk}}t_{\text{phase}} \right)}\sum_{k} A_{\text{ASE},k}e^{-b{{\Delta D}_{k}\left( \hat{g}\cdot\hat{n}_{k} \right)}^{2}}e^{i\omega_{\text{myelin,k}}t_{\text{phase}}},$ (11)

where we sum over multiple crossing fibre populations $k$.

The parameters in this equation are:

- Acquisition parameters
  - $b$: quantifies the sensitivity to the diffusivity
  - $\hat{g}$: orientation of the diffusion-weighted gradient
  - $t_{\text{phase}}$: phase accumulation time between the second spin echo and the centre of the second readout
- Free parameters fitting the signal magnitude
  - $A_{\text{SE},k}$: signal amplitude perpendicular to the fibre orientation at the first readout
  - ${\Delta D}_{k}$: signal width corresponding to parallel minus perpendicular apparent diffusivity
  - $\hat{n}_{k}$: average fibre orientation
  - $A_{\text{ASE},k}$: signal amplitude perpendicular to the fibre orientation at the second (asymmetric) readout, which is fitted independently for each $t_{\text{phase}}$. With multiple $t_{\text{phase}}$ can be used to estimate $T_{2;k}$ and $T_{2;k}^{*}$.
- Free parameters fitting the signal phase
  - $\phi_{\text{SE}}$: any phase accumulated before the first readout. This is estimated independently for each volume.
  - ${\Delta\phi}_{\text{eddy}}$: eddy-current induced phase offset between the two readouts, which is modelled as a function of gradient orientation using spherical harmonics (equation 4). Odd and even components of the spherical harmonics are treated differently:
    - Odd components of the spherical harmonics are estimated independently for each $t_{\text{phase}}$
    - Even components are either assumed to be 0 or if only data with $t_{\text{phase}}>0$ is acquired or constant across all $t_{\text{phase}}$ if data with $t_{\text{phase}}=0$ is also acquired, effectively setting them to the value estimated at $t_{\text{phase}}=0$. Any more realistic model that allows for variability with $t_{\text{phase}}$ will lead to degeneracies with the estimated *g*-ratio
  - $\omega_{\text{bulk}}$: off-resonance frequency due to non-myelin sources. Assumed to be a constant in each voxel (i.e., does not depend on fibre population or any of the acquisition parameters)
  - $\omega_{\text{myelin};k}$: myelin-induced frequency offset. Can be used to estimate $\left\langle\log g \right\rangle_{k}$ under the assumption that the signal is dominated by intra-axonal water. If only short $t_{\text{phase}}$ are acquired, can be related to the average log *g*-ratio ($\left\langle\log g \right\rangle_{k}$) through equation 7. Alternatively, a two-compartment fit (equation 8) can be applied if multiple including long $t_{\text{phase}}$ were acquired. The latter estimates both the signal fraction ($f_{\text{myelin;}\text{k}}$) and log *g*-ratio ($\left\langle\log g \right\rangle_{\text{myelin};k}$ ) of the myelinated axons.

Table S1 Acquisition requirements for the parameters of interest

| Acquisition (all single b-value) | | What can be estimated |
| --- | --- | --- |
| Head orientations | $\boldsymbol{t}_{\text{phase}}$ |  |
| Single | Single (non-zero) | $\left\langle\log g_{1} \right\rangle\sin^{2} \theta_{1}-\left\langle\log g_{2} \right\rangle\sin^{2} \theta_{2}$ |
| Multiple | Single (non-zero) | $\left\langle\log g \right\rangle_{k}$ per fibre population |
| Multiple | Multiple (non-zero) | $f_{\text{myelin;}\text{k}}$ and $\left\langle\log g \right\rangle_{\text{myelin};k}$ instead of $\left\langle\log g \right\rangle_{k}$ |
| Single or Multiple | Any above *and* $\boldsymbol{t}_{\text{phase}}\boldsymbol{=0}$ | Also: $T_{2,k}$, $T_{2,k}^{'}$, $\phi_{\text{eddy}, \text{sym}}(t_{\text{phase}}=0)$ |

## S2: Parameter fitting

Parameter estimation is complicated by the phase wrapping inherent in complex MRI data. The signal is identical for a phase of $\phi$ and $\phi+2\pi n$ for any integer $n$, which leads to many unphysical, local minima when fitting the phase. While phase unwrapping^56^ could potentially deal with this by estimating $n$ for each gradient orientation relative to the others, such unwrapping is complicated by the low SNR inherent in diffusion MRI for gradient orientations aligned with the dominant fibre orientation. Instead, we propose to deal with the phase wraps through a careful initialisation of the parameters and a multi-step fitting procedure. At each step the results of the previous fit are used to initialise the new fit:

1. In actual DIPPI data the number of fibres and their orientations can be estimated from any model allowing for crossing fibres such as ball & stick^29^ or constrained spherical deconvolution^57^. In this work, we only simulate data with two crossing fibres and then fit it assuming two crossing fibres. Fibre orientations are initialised in this work to the first two eigenvectors of a diffusion tensor fit.
2. In the initial part of the fitting we only fit the signal magnitude. First, we just fit the amplitudes ($A_{\text{SE},k}$ and $A_{\text{A}\text{SE},k}$ or $T_{2;k}$ and $T_{2;k}^{*}$), then we fit the amplitudes and width, and finally the amplitude, width and orientation (i.e., the full Watson distribution).
3. Once we have a decent fit for the signal magnitude, we can estimate the parameters influencing the signal phase:
   1. The phase offsets induced during the diffusion encoding ($\phi_{\text{SE}}$) are initialised by the phase measured during the first readout. While such direct phase estimates can be very noisy for gradient orientations with very low SNR ($\lesssim1$), any gradient orientations with such low SNR at the first readout will be so dominated by noise at the second readout that they do not contribute significantly to the final fit.
   2. The $l=1$ spherical harmonic components of the eddy currents across gradient orientations are then estimated for each $t_{\text{phase}}$ through the following algorithm:
      1. We compute a convex hull containing all the gradient orientations using the Quickhull algorithm^58^ from [www.quickhull.org](http://www.quickhull.org). Any gradient orientations connected in this hull are considered neighbours.
      2. For each pair of neighbouring gradient orientations the phase difference is computed (and mapped between $-\pi$ and $\pi$ by adding or subtracting $2\pi$).
      3. The linear phase gradient in the x-direction $G_{x}$ is then estimated by solving: $\text{min}_{G_{x}}\sum_{i} m_{i}^{2}\left( G_{x}\Delta g_{x;i}-\Delta\phi_{i} \right)^{2},$where the sum is over each pair of neighbours $i$, $\Delta\phi_{i}$ is the phase difference computed in step 2, $m_{i}$ is the average magnitude of the signal for the neighbouring gradient orientations, and $\Delta g_{x;i}$ is the difference in the x-component of the gradient between the neighbours. The same equation is solved for the y- and z-components. The phase gradient estimates are multiplied by$\sqrt{\frac{4\pi}{3}}$ to get the first-order spherical harmonic components.
   3. The non-myelin off-resonance frequency ($\omega_{\text{bulk}}$) and *g*-ratio parameters are randomly initialised. $\omega_{\text{bulk}}$ is drawn from a uniform distribution between -300 and 300 Hz, which is a substantially larger range than seen in susceptibility-weighted MRI. For a single-population model the $\left\langle\log g \right\rangle_{k}$ is initialised randomly between log 0.6 and log 1 (= 0). For the two-population model $f_{\text{myelin;}\text{k}}$ is initialised randomly between 0 and 1 and $\left\langle\log g \right\rangle_{k}$ between log 0.6 and log 0.8.
   4. We then fit in order just the non-myelin off-resonance frequency ($\omega_{\text{other}}$), the non-myelin off-resonance frequency and the spherical harmonic components of the eddy currents, and finally also include the *g*-ratio parameters (average log *g*-ratio and $f_{\text{myelin;}\text{k}}$).
   5. Steps c-d are repeated until the global minimum is found
4. Finally, we include a fit including all free parameters (both phase- and amplitude-related as listed in S1) initialised from the values found above.

All fits were carried out in python using local optimisation with the quasi-Newton method L-BFGS-B^59,60^ from the scipy library with gradients computed symbolically using the sympy library.

## S3: Unwrapping the phase on a sphere

Another way to avoid the local minima when fitting phase data as discussed in S2 is to unwrap the phase of the data before fitting. Note that as opposed to the more commonly spatial phase unwrapping across an image^56^, we apply phase unwrapping here across the gradient orientations within each voxel.

Similarly, to the estimation of the $l=1$ components in S2 we start by defining neighbouring gradient orientations as those connected in a convex hull^58^. Starting from some random gradient orientations, any phase wraps in the neighbouring gradients are corrected by subtracting or adding $2\pi$ to their phases. For each of the neighbours the algorithm is then repeated and so on, until all the phases for all gradient orientations have been unwrapped.

This approach is only expected to work if the SNR is consistently high enough to produce reliable phase estimates for all gradient orientations. This is the case for the phantom data, where we apply phase unwrapping, however it will not generally be the case for DIPPI data, which is why we do not propose to use phase unwrapping when fitting the DIPPI model (where instead we fit directly to the complex data). This phase unwrapping algorithm could probably be made more accurate by adopting some of the techniques used in Jezzard and Balaban (1995)^55^, but we do not explore that here.
